# Fatty acid metabolism in gouty arthritis: mechanisms to therapeutic targeting

**DOI:** 10.3389/fimmu.2025.1671548

**Published:** 2025-10-16

**Authors:** Xueping Zhao, Ye Sun, Le Yang, Hui Sun, Xinya Zhang, Hui Sun, Guangli Yan, Xijun Wang

**Affiliations:** ^1^ State Key Laboratory of Dampness Syndrome of Chinese Medicine, The Second Affiliated Hospital of Guangzhou University of Chinese Medicine, Guangzhou, China; ^2^ State Key Laboratory of Integration and Innovation for Classic Formula and Modern Chinese Medicine, National Chinmedomics Research Center, Metabolomics Laboratory, Department of Pharmaceutical Analysis, Heilongjiang University of Chinese Medicine, Harbin, China

**Keywords:** macrophages, neutrophil, fatty acid, gouty arthritis, monosodium urate, fatty acid metabolism

## Abstract

Gouty arthritis (GA), a condition characterized by monosodium urate (MSU) crystal deposition and NLRP3 inflammasome-driven inflammation, is a result of a complex interplay between hyperuricemia and immune dysregulation, which leads to systemic complications and joint damage. Current therapies for GA exhibit certain limitations, including cardiovascular risks, hepatotoxicity, low efficacy in special populations, and difficulty in dissolving tophi. Emerging evidence implicates fatty acid metabolism disorders as key pathogenic factors in GA. Elevated fatty acids (FAs) activate Toll-like receptors (TLRs) in macrophages, which act in synergy with MSU crystals to trigger NLRP3 inflammasome activation and pro-inflammatory cytokine release (e.g., IL-1β), thereby initiating the inflammatory cascade. Dysregulated FA metabolism promotes neutrophil recruitment through aberrant arachidonic acid (AA) metabolism and exacerbates hyperuricemia by increasing purine synthesis while inhibiting uric acid excretion. Consequently, future clinical practice may leverage the detection of FA signatures in GA patients to enable tailored therapeutic and dietary management, thereby maximizing treatment efficacy while minimizing adverse effects. The combined application of FA-modulating agents and anti-GA therapeutics synergistically enhances therapeutic efficacy, enabling comprehensive disease-modifying control over GA progression. This review systematically elucidates the mechanisms through which FA metabolism disorders drive the progression of GA, providing a scientific basis for the subsequent research on GA.

## Graphical Abstract

FA, fatty acid; MSU, monosodium urate; TG, triglyceride; GLUT9, glucose transporter type 9; URAT1, urate trans**p**orter 1; AA, arachidonic acid; IL-1β, interleukin - 1 beta; TLR, Toll-like receptor; TNF-α, tumor necrosis factor-alpha.

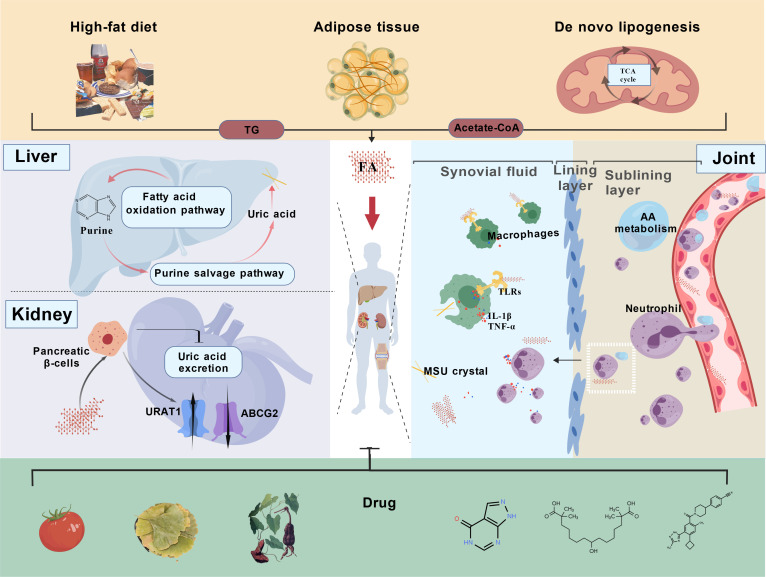

## 1 Introduction

Gouty arthritis (GA) is one of the most common types of inflammatory arthritis (1), characterized by the deposition of monosodium urate (MSU) crystals in joints and surrounding tissues under conditions of persistent hyperuricemia (2), which activates the innate immune system and triggers an inflammatory response. It is a disease caused by metabolic disorders (3). The global prevalence of GA ranges from 0.68% to 3.90%, increasing the burden on healthcare (4, 5). High uric acid levels alone are not a sufficient condition for the onset of GA: follow-up studies have shown that some individuals with low uric acid levels develop GA, while approximately 50% of individuals with high uric acid levels do not develop the condition during a 15-year follow-up period (6, 7). This contradictory clinical presentation poses a challenge for both clinicians and basic scientists.

MSU crystals were previously thought to be an endogenous danger signal for GA. However, most *in vitro* studies have identified MSU crystals as inflammasome activators needed to use lipopolysaccharide (LPS) or phorbol-12-myristate-13-acetate (PMA) to trigger the activation of the inflammasome in cells (8–11). Moreover, a comprehensive analysis of the data from 268,174 participants in the UK Biobank revealed that the plasma FA levels of 5,160 participants who developed GA were associated with the risk of GA onset (12). Another relevant study reported similar results: serum FA levels were significantly higher in patients with acute GA than in those with GA in remission, asymptomatic hyperuricemic patients, and normal controls (13). These findings suggest that FAs serve as important cofactors in MSU-induced inflammation. Other factors may act synergistically to trigger inflammation, and FA may be one of the key factors.

Further exploration revealed that during the course of inflammation, FA-related metabolic disorders promote inflammation by affecting the metabolism and microenvironment of macrophages and neutrophils (14–16). In addition, FA may aggravate hyperuricemia by promoting purine synthesis and inhibiting uric acid excretion (17–19). Traditional studies on GA and FAs, such as TAG, DAG, and PC, have focused primarily on lipid changes. However, this review uniquely bridges the knowledge gap in prior literature by elucidating the relationship between fatty acid metabolism and GA through a tripartite analysis of fatty acid synthesis, degradation pathways, and free FA levels. Moreover, fatty acid metabolism exerts distinct roles in GA, exemplified by its promotion of M1 macrophage polarization via fatty acid oxidation—a mechanism that operates conversely in other diseases. Given the strong associations between fatty acid metabolism and immune responses and elevated uric acid levels during GA attacks, the progression of GA under the mediation of fatty acid metabolic disorders was reviewed in this paper to provide a theoretical basis for exploring novel therapies for GA.

## 2 Comprehensive overview of fatty acids

FAs are organic acids that are defined mainly by the length and saturation of their aliphatic side chain. When classified according to the length of their side chain, FAs are divided into three types. Short-chain FAs (SCFAs), also known as volatile FAs, have 2 to 6 carbon atoms within their structures. FAs with chain lengths of 6 to 11 carbon atoms are called medium-chain fatty acids (MCFAs), whereas FAs with structures comprising alkyl chain lengths greater than 12 carbon atoms are considered long-chain fatty acids (LCFAs). LCFAs are usually present in vegetable oils, animal fats, or marine oils (20) and include palmitic acid (C16:0), palmitoleic acid (C16:1), arachidonic acid (20:4n-6), and docosahexaenoic acid (22:6n-3). C16-C18FAs are components of FA-derived signaling molecules. Long-chain polyunsaturated FAs such as arachidonic acid (AA) and docosahexaenoic acid serve as precursors of the major lipid signaling molecules prostaglandin (PEG2) and leukotriene (LT) (16). FAs also serve as important nutrients for the human body. The intake, transport, and metabolism of FAs in the body are complex and intricate processes.

### 2.1 Free fatty acid intake and transport

FA is ingested mainly from food in the form of triglycerides (TGs). TGs are formed by the combination of three FA molecules with one glycerol molecule and exist primarily in the form of dietary fat (about 95%) (21). TG is naturally hydrophobic. In the oral cavity, stomach, and intestinal cavity, TG is hydrolyzed by various lipases to produce two FA molecules and one monoacylglycerol (MAG) molecule. SCFAs (containing 2–6 carbon atoms) and MCFAs in the hydrolysis products directly enter the portal vein and are then transported to the liver through the bloodstream. The absorbed LCFA are resynthesized into TG in the small intestinal mucosal cells and form chylomicrons with apolipoproteins, cholesterol, etc (22). Subsequently, these chylomicrons are transported into the plasma through the lymphatic system, and most of the TG within these chylomicrons is absorbed by various tissues through the circulatory system (23).

### 2.2 Fatty acid metabolism

After cellular uptake, FAs are degraded mainly through mitochondrial FA β-oxidation, which is crucial for maintaining energy homeostasis in the human body (24). FAs bind to coenzyme A (CoA) in the cytoplasm to form acyl-CoA. Long-chain acyl-CoA then enters the mitochondria under the action of carnitine palmitoyltransferase 1 (CPT1), where it is converted to fatty acyl-carnitine (25). The β-oxidation process breaks down FAs into acetyl-CoA, which is then utilized in the mitochondrial tricarboxylic acid (TCA) cycle to produce adenosine triphosphate (ATP) for energy (26, 27). However, when FA levels exceed the energy requirements of the cells, these FAs are stored in the adipose tissue mainly in the form of TG. In periods of energy deficiency, TG hydrolysis occurs, producing FA and glycerol, and in this process of fat breakdown, energy is released for internal use. In addition, FAs enter the vascular system for use as an energy substrate in other organs (28). TG is hydrolyzed sequentially to form diacylglycerol (DAG) and then MAG, releasing FA at each stage. MAG is hydrolyzed to release the final FA and glycerol. The production of FA and glycerol from stored TG utilizes a series of highly coordinated enzymatic actions involving adipose triglyceride lipase (TGL), hormone-sensitive lipase (HSL), and monoacylglycerol lipase (MGL) (29).

In the condition of an imbalance of the production and degradation of FAs, the levels of circulating FAs in the body increase. This paper focuses on the role of elevated circulating FA levels in the pathogenesis of GA.

## 3 Fatty acid biomarkers for gout arthritis

A metabolomics study based on serum NMR spectroscopy of asymptomatic hyperuricemic and GA patients revealed significantly altered FA levels, which may serve as risk biomarkers for disease onset (30). A study that used lipidomics to distinguish early-onset hyperuricemia from GA revealed a systemic elevation in FA levels among patients with hyperuricemia and GA. Shen et al. identified dysregulated pathways and potential metabolic biomarkers for hyperuricemia and GA using serum metabolomics. The authors discovered that AA can be used as a marker to distinguish hyperuricemic patients from non-hyperuricemic individuals. Furthermore, the authors identified two fatty acids – AA and myristic acid – as potential biomarkers for differentiating GA patients from hyperuricemic patients (31). Moreover, Wang et al. reported that arachidic acid can be used as a metabolic marker to distinguish frequent GA episodes from infrequent GA episodes, thereby representing a potential biomarker. Furthermore, eicosapentaenoic acid levels are significantly lower in patients with frequent seizures than in those with infrequent seizures (32). A cohort study involving the integrated use of genetic susceptibility and metabolomics along with 1,708 GA cases and a follow-up period of 9.47 years to investigate the association between dietary polyunsaturated FAs and GA risk reported that PUFA, n-6 PUFA, n-3 PUFA, ALA, and EPA intake were negatively associated with gout risk, and AA intake was positively associated with gout risk (33). Wang et al. reported that stearic acid concentrations were reduced in acute GA patients and could potentially serve as a specific biomarker for acute GA (34). A comprehensive analysis of a population cohort and genetic data of 15,194 participants revealed that SFAs, MUFAs, n-3 PUFAs, and docosahexaenoic acid (DHA) were positively associated with GA events (P values < 0.0001), while PUFAs, n-6 PUFAs, and linoleic acid were negatively associated with GA incidence (all trend P values < 0.0001) (12). However, a study on dietary supplementation with n-3 PUFAs polyunsaturated FAs indicated that the intake of n-3 PUFAs, which are abundant in dietary fish, is associated with a lower risk of GA recurrence (35, 36). Therefore, the role of n-6 and n-3 PUFAs in the GA still requires further validation.

Clinical omics research has concluded that abnormal changes in FA levels play a pivotal role in the progression of gestational age (GA). However, the current studies on GA have not extensively investigated the role of unsaturated FAs in regulating the GA pathway, and the existing research remains incomplete. Pandey, S. proposed that the research on the treatment for immune-mediated diseases, such as rheumatoid arthritis, should attempt to achieve a more comprehensive understanding of various disease-related biomarkers, conducting large-scale high-throughput data analysis, and identifying and quantifying the disease metabolites (37). Therefore, subsequent research on GA can comprehensively analyze its lipid biomarkers through lipidomics, providing a basis for both fundamental research on GA and its precise clinical application.

## 4 Disorders of fatty acid metabolism promote the development of gouty arthritis

**Figure 1:**
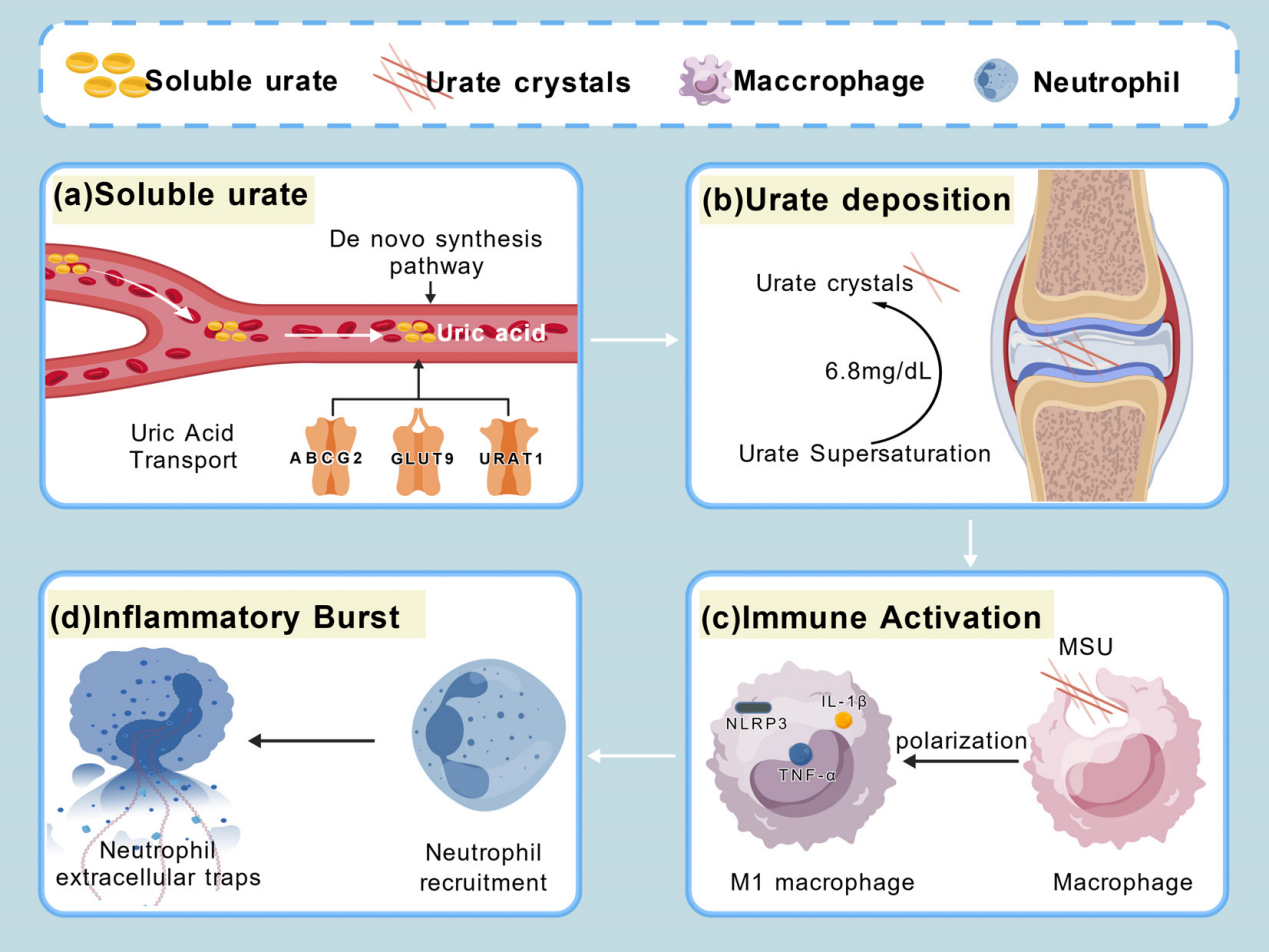
**(a)** Imbalance between uric acid production and transport leads to elevated uric acid levels. **(b)** Uric acid levels exceeding 6.8 mg/dL result in the precipitation of urate crystals. **(c)** Macrophages phagocytose MSU crystals to activate immune responses. **(d)** Macrophages are recruited to the site of injury and release neutrophil extracellular traps.

A diagram illustrating the relationship between gouty arthritis and immunity (**Figure 1**).

### 4.1 Macrophage fatty acid metabolic disorders potentiate MSU crystal-induced inflammation

Numerous studies have shown that the inflammatory factor interleukin-1 beta (IL-1β) is essential for MSU crystal-driven inflammation and is released, in place of infiltrating neutrophils or monocytes, from the resident macrophages in the initial stage of GA (38, 39). However, the production of IL-1β during the GA process is puzzling, with studies reporting that MSU crystals alone do not participate in the transcription of IL-1β but only in the regulation of the conversion of inactive IL-1β to an activated state (10, 11). Post-transcriptional IL-1β exists solely in the inactive Pro-IL-1β form, which has to be cleaved by the NLRP3 inflammasome-activated effector protein caspase-1 for activation, and this activated form then exerts the proinflammatory effects (40).

Joosten et al. conducted an in-depth study and reported that FAs participate in the transcription of NLRP3 and Pro-IL-1β, promoting the onset of GA inflammation by activating the Toll-like receptor (TLR) pathway in conjunction with MSU (41). Mechanistically, the TLR2/TLR4 proteins, which are members of the TLR family and are highly expressed in the synovial macrophages of GA patients, are first activated through the recognition of FAs. TLRs are pattern recognition receptors (PRRs) involved in the innate immune system and can recognize pathogen-associated molecular patterns (PAMPs) and endogenous ligands (41–44). Another perspective is that fatty acid synthesis may promote inflammation by facilitating TLR activation (45). The palmitate produced through the FASN pathway can be reversibly conjugated to the cysteine residues of target proteins in a process known as palmitoylation (S-acylation) (46). The palmitoylation process affects TLR/MYD88 signaling, and FASN supplies the endogenous FAs required for MYD88 palmitoylation, with cysteine 113 serving as a critical site for MYD88 palmitoylation. This process stabilizes the intermediate domain, which ensures the binding of MYD88 to interleukin-1 receptor-associated kinase 4 (IRAK4). IRAK4 is a pivotal molecule linking the TLR receptors to downstream inflammatory signaling activation, thereby initiating the TLR downstream signaling cascade (45). Studies have indicated that fatty acid synthesis enzyme (FASN) influences subsequent inflammatory development by regulating the TLR signaling pathways. FASN is a multidomain enzyme with a ketosynthetase domain that processes acetoacetyl-CoA, an intermediate metabolite that serves as a key substrate for lipid rafts. The activation of FASN increases the production of acetoacetyl-CoA, significantly altering lipid raft composition (47). Inhibition of FASN or acetoacetyl-CoA effectively reduces the affinity between the lipid rafts and TLR4, thereby diminishing the migration of TLR4 toward the lipid rafts, preventing its specific recognition function (47) Therefore, activated FASN may significantly increase the ability of TLR4 to recognize ligands.

Thus, TLR2/TLR4 activation promotes the dissociation of the inhibitor of nuclear factor kappa-B (I-κB)/NF-κB complex through a myeloid differentiation primary response 88-dependent (MyD88) signaling pathway, mediating the nuclear translocation of nuclear factor kappaB (NF-κB) and specifically acting on the NLRP3 and IL-1B gene promoter regions. This leads to the mediation of the transcription of NLRP3 and pro-IL-1β, resulting in the production of many inactive inflammatory mediators, leading to subsequent protease cleavage and activation (48–51). TLR4 can also form a TLR4/myeloid differentiation factor 2 (MD2) complex to further mediate the signal transduction (52). Finally, inactive NLRP3 and pro-IL-1β mediate the initiation of inflammation upon stimulation by MSU crystals. After the macrophages ingest MSU through the phagocytic pathway, NLRP3 inflammasome assembly and activation are triggered, and the generated complex consists of NLRP3, the adaptor protein apoptosis-related cyclin-like protein (ASC), and caspase-1 (53). The activation of this complex induces the self-cleavage of pro-caspase-1 into active caspase-1, which then undergoes a site-specific hydrolysis of pro-IL-1β to generate mature IL-1β, thereby activating the related signaling pathways to mediate the inflammatory cascades (40, 54). Moreover, Leishman et al. proposed that fatty acid synthesis pathways play crucial roles in NLRP3 inflammasome assembly and activation by palmitoylating NLRP3, during the initiation step, at the Cys898 site of NLRP3 (55). Subsequently, palmitoylation mediates the process of NLRP3 accumulation within the endosome, facilitating its binding to the dispersed trans-Golgi network vesicles for their complete assembly (55, 56). On the basis of the above mechanism, it can be inferred that fatty acid metabolism may play an important role in the initiation of inflammation in GA macrophages. Single MSU crystal phagocytosis has a limited effect on the production of large amounts of inflammatory factors, and the FA-mediated TLR/MyD88 pathway plays a key role in this process.

FA can also induce the direct activation of the NLRP3 inflammasome in macrophages. Palmitate can activate the NLRP3 inflammasome through lysosomal instability in macrophages (57). In addition, palmitate inhibits the phosphorylation of adenosine 5denosinelation_ENREF_57” protein kinase (AMPK) and blocks autophagy, leading to increased ROS levels in macrophages, which in turn activate the NLRP3 inflammasome and IL-1β secretion (58).

On the other hand, macrophages respond to various stimuli in the microenvironment and are reprogrammable into different functional subtypes after activation, usually converting in two directions: pro-inflammatory and anti-inflammatory subtypes, which are not fixed states but are mutually convertible for suitability to the changing needs and environments (59). In a gout zebrafish model within a complete microenvironment, macrophage-dependent fatty acid oxidation (FAO) promoted the conversion of the proinflammatory phenotype (60). FAO-driven mitochondrial-derived ROS (mROS) depend on the expression of immune-responsive gene 1 (irg1) (61), which has been identified as one of the highly overexpressed genes in acute GA macrophages (62). MSU crystal-activated irg1 drives mROS to promote macrophage IL-1β and tumor necrosis factor-alpha (TNF-α) expression. Many previous studies have suggested that FA is the main metabolic pattern of anti-inflammatory macrophages, whereas aerobic glycolysis is considered the driving factor of pro-inflammatory states (27). However, most studies on macrophage metabolic reprogramming have remained limited to the use of *in vitro* techniques utilizing few inflammatory stimuli (mainly LPS) or non-GA models, and are, therefore, inadequate for explaining macrophage polarization in GA (63). Jiang et al. explored this topic and reported that inhibiting FAO can suppress the inflammatory response of macrophages in MSU crystal-induced GA models, providing evidence supporting the above view (64). Liu et al. reported that suppressing AA metabolism by using COX-2, 5-LOX, and CYP4A in macrophages under GA conditions shifted the macrophages away from the M1 phenotype (65). On the basis of the above explanation, it is possible that the metabolic pattern of macrophages relies highly on changes in the intact microenvironment and that FAO in the GA model drives macrophages toward a proinflammatory phenotype.

**Figure 2:**
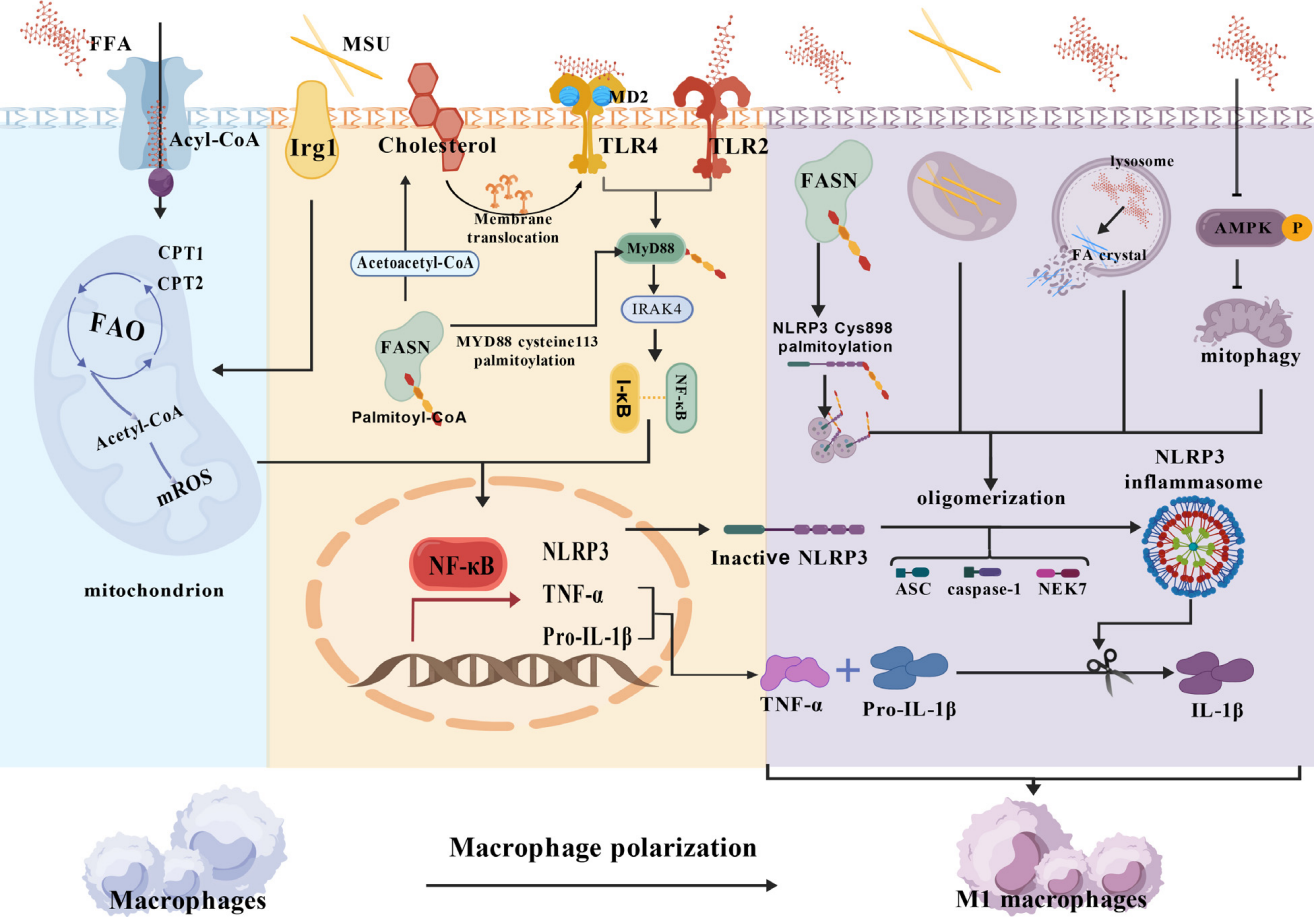
Macrophage fatty acid metabolic disorder potentiates MSU crystal-induced inflammation. [Created with BioGDP.com (244)] NLRP3, Nucleotide-binding oligomerization domain-like receptor family, pyrin domain-containing 3; ASC, adaptor protein apoptosis-related cyclin-like protein; FAO, Fatty acid oxidation; CPT1,2, Carnitine palmitoyltransferase 1,2; Irg1, Immune-responsive gene 1; MyD88, Myeloid differentiation primary response 88; I-κB, inhibitor of nuclear factor kappa-B; MD2, Myeloid differentiation factor 2;AMPK, adenosine 5′-monophosphate-activated protein kinase; NF-κB, Nuclear factor kappaB; FASN, Fatty Acid Synthase; (1) FFA promotes Pro-IL-1β, TNF-α, and NLRP3 transcription via FAO, FFA/TLRs; (2) FFA synergizes with MSU crystals to activate NLRP3 inflammatory vesicles via AMPK/ROS/NLRP3, lysosomal instability/NLRP3 pathways, promotes IL-1β release, and promotes macrophage M2 polarization.3.2 Disorders of fatty acid metabolism: promotion of neutrophil recruitment.

In summary, a large body of research evidence shows that FA synergizes with MSU crystals to induce the initiation of inflammation in GA: FA induces macrophage activation by activating TLR receptors, lysosomal instability/NLRP3, and AMPK/ROS/NLRP3 pathways, while more active FAO promotes the conversion of macrophages to a pro-inflammatory phenotype (**Figure 2**).

### 4.2 Fatty acid metabolic dysregulation promotes neutrophil recruitment in gouty arthritis

Neutrophils are the most abundant white blood cells in human peripheral blood. These cells are rapidly recruited from the blood to the inflamed tissues under the action of chemokines that signal danger (66). Acute inflammation in GA is accompanied by neutrophil infiltration, which can promote this process by stimulating the macrophages to produce inflammatory factors and chemokines (67, 68). Furthermore, studies have shown that the FA metabolites in neutrophils play a chemotactic role in the infiltration of neutrophils into the inflammatory sites, with AA metabolism playing a dominant role (69, 70) Multiple clinical studies have revealed that patients with GA exhibit abnormal AA metabolism, significantly elevated 5-LOX transcription levels, and abnormal increases in LTB4 production, which acts as a neutrophil chemotactic factor (71–73). AA is broken down into different metabolites through three metabolic pathways: lipoxygenase, cytochrome P450 monooxygenase, and cyclooxygenase. LTB4 is produced through the lipoxygenase pathway and exerts its effects (74).

Rae et al. conducted clinical research and reported that MSU not only stimulates white blood cells to produce more LTB4 but also inhibits LTB4 metabolism, causing it to persist and recruit more neutrophils (75). Subsequent studies by other researchers investigated the treatment of GA by inhibiting the LTB4 production pathway, and the results further corroborated the above conclusion (61). Although the mechanism through which MSU crystals promote AA metabolism toward LTB4 production remains unclear to date, the mechanism through which MSU crystals recruit neutrophils to the sites of inflammation is relatively well understood. To elaborate, after responding to MSU crystal stimulation and producing primary chemotactic signals, neutrophils release LTB4 through autocrine and paracrine mechanisms and form chemotactic gradients to recruit cells from a distance, causing a significant increase in the range and persistence of detection (76–78). In the MSU-induced GA mouse model, complement C5a was secreted as a primary chemotactic signal for neutrophils (77), which could then bind to the G protein-coupled receptors to generate calcium ion flux (79), thereby mediating vesicle fusion with the cell membrane and promoting the secretion of exosomes coated with LTB4 (80). During exosome formation, MSU crystal-stimulated neutrophils promote the synthesis of LTB4: cytosolic phospholipase A2 alpha translocates to the nuclear membrane, where it releases AA from membrane-bound phospholipids, whereas 5-lipoxygenase (5-LOX) is mobilized to the nuclear membrane, where it binds to 5-LOX-activating protein (FLAP) and acts on AA to produce leukotriene A4 (LTA4). Proteomic and metabolomic studies revealed that the levels of phospholipase A2 in the synovial fluid of GA patients are significantly elevated and exhibit a strong interaction with the significantly increased metabolite PE (81). Simultaneously, 5-LOX is recruited to the nuclear envelope, where it binds to the 5-LOX-activating protein (FLAP) and acts on AA to generate leukotriene A4 (LTA4). LTA4 is ultimately converted, under the action of LTA4 hydrolase (LTA4H), into LTB4, which is encapsulated within exosomes for release (82).

The subsequently released LTB4 causes neutrophils to adhere to and become trapped within the blood vessels, leading to their extravasation and migration from the bloodstream into the inflamed tissue. The process involves multiple steps and fine regulation, with LTB4 signal transduction coordinating the dynamic redistribution of non-muscle myosin IIA (NMIIA) and β2-integrin (Itgb2), thereby promoting neutrophil arrest and extravasation. First, leukotriene B4 receptor 1 (BLT1) is a chemotactic G protein-coupled receptor expressed by leukocytes (83). LTB4 binds to BLT1 to activate leukocytes and prolong their survival time (84). The LTB4-BLT1 axis is essential for the sustained recruitment, stasis, and extravasation of neutrophils (85, 86). The LTB4-BLT1 axis subsequently promotes the redistribution of NMIIA from the cytoplasm to the cell cortex by regulating its kinetics, thereby providing the contractile force required for neutrophils to squeeze through the exudation site (85). Ultimately, the activated NMIIA continues to act on its downstream target, Itgb2, regulating its kinetics and localization on the plasma membrane (PM) to promote the stasis of rigid neutrophils (87). Abnormal AA metabolism disorders in GA promote the production of the neutrophil chemokine LTB4 and mediate the migration of neutrophils to the inflammatory sites through paracrine and autocrine mechanisms.

Additionally, the neutrophils arriving at the site of injury can trigger the release of inflammatory cytokines by releasing neutrophil extracellular traps (NETs), thereby promoting the inflammatory response (88). LTB4 acts as a key signaling molecule, coordinating neutrophil activation and guiding the formation of NETs (89). Research has demonstrated that saturated FAs promote the release of NETs through the TLR4-MD2/ROS signaling pathway (90) Neutrophil extracellular traps (NETs) are fibrous web-like structures that protrude from the membranes of activated neutrophils and are coated with histones, proteases, and granular cytosolic proteins (91). SFA induces the formation of the TLR4-MD2 complex, which further promotes NOX-derived ROS production. The generation of ROS serves as the basis for NET formation, thereby exacerbating NET release (90). Moreover, FAs activate NADPH oxidase to promote ROS production and increase the expression of p38, ERK, and JNK. The massive release of ROS and the activation of the JNK and ERK pathways induce the formation of NETs; however, the specific mechanism remains unclear (92).

In addition, studies have shown that dietary SFAs play a powerful role in promoting the transport of neutrophils from the bone marrow to the blood through the C-X-C motif chemokine ligand 2/C-X-C motif chemokine receptor 2 axis (14). The amplification of neutrophil purinergic activity during migration depends on Cpt1A-mediated FAO. Cpt1A is considered the rate-limiting enzyme in the LCFA mitochondrial metabolism, and its inhibition reportedly suppresses the purinergic transfer in neutrophils, reducing their chemotactic ability (93). Immature neutrophils also rely on mitochondrial FA β-oxidation for ATP. In experimental studies, when Mycobacterium tuberculosis (Mtb) infection induced neutrophil recruitment to the site of infection, immature neutrophils took in exogenous FA to obtain energy to reach the site of infection (94).

**Figure 3:**
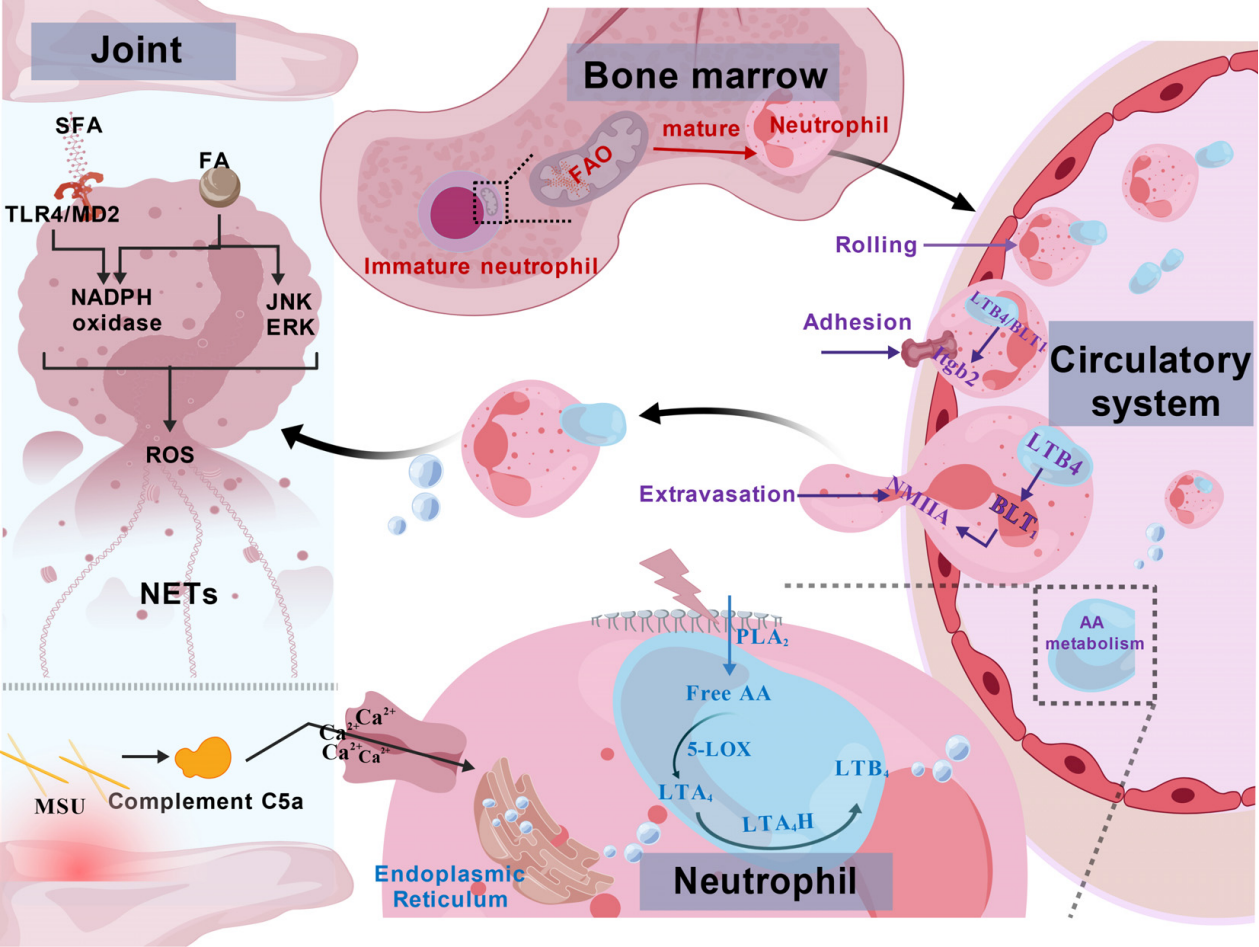
Fatty acid metabolic dysregulation promotes neutrophil recruitment in gouty arthritis. LTB4, Leukotriene B4; NMIIA, non-muscle myosin IIA; Itgb2, β2-integrin; LTA_4_, leukotriene A4; LTA_4_H, LTA4 hydrolase; PLA_2_, Phospholipase A_2_; 5-LOX, 5 – Lipoxygenase; NETs, Neutrophil Extracellular Traps; JNK, c-Jun N-terminal Kinase; ERK, Extracellular Signal-Regulated Kinase; SFA, Saturated Fatty Acids. (1) Immature neutrophils are dependent on FAO for energy supply; (2) the GA environment activates AA metabolism to produce LTB4 to form a chemotactic gradient that causes neutrophils to roll, adhere, extravasate, and recruit to the site of infection in the vasculature.

In summary, during acute GA attacks, the migration of neutrophils and the promotion of inflammatory processes are closely linked to FA metabolism (**Figure 3**).

### 4.3 Fatty acid metabolic dysregulation induces hyperuricemia in gout pathogenesis

Hyperuricemia is an established modifiable risk factor for gouty arthritis (GA), which directly influences disease progression through dynamic fluctuations in serum urate levels (95). FAs derived from dietary fat intake, adipose tissue lipolysis, and *de novo* fatty acid synthesis (DNL) induce purine salvage synthesis pathways to promote excessive uric acid synthesis (18, 96–99). Most people with endogenous excess uric acid production appear to have a salvage pathway, and FA promotes uric acid synthesis through the purine salvage pathway by activating hypoxia-inducible factor-1α (HIF-1α) to upregulate the expression of purine metabolism-related enzymes (18, 100–102).

HIF-1α serves as the primary molecular mediator of the hypoxic response. The single-cell transcriptomic analysis of GA patients revealed that the encoding gene, HIF-1A, is significantly upregulated in the monocytes of GA patients (103). This upregulation occurs under hypoxic conditions, leading to its binding to HIF-1β within the cell nucleus, where HIF-1α interacts with hypoxia response elements (HREs) to induce the activation of numerous downstream genes (104).

FAs can increase the *de novo* synthesis of HIF-1α at the translational level through fatty acid-binding protein 5 (FABP5) (105), and in GA models, the FA-induced significant upregulation of acyl-CoA synthase long-chain family member 1 (ACSL1) and CPT1A-promoted FA β-oxidation together activate HIF-1α in the liver cells (106). Activated HIF-1α enters the cell nucleus, where it binds to the promoters of the xanthine dehydrogenase (XDH) and 5’-nucleotidase II (NT5C2) genes, thereby upregulating the expression of XDH and NT5C2. NT5C2 is the enzyme responsible for catalyzing the hydrolysis of inosine monophosphate (IMP) and guanosine monophosphate (GMP). After hydrolysis, IMP and GMP are converted, under the action of other enzymes, to hypoxanthine and xanthine, which serve as substrates for XDH, thereby increasing the consumption of hypoxanthine and the production of uric acid in the liver (107).

**Figure 4:**
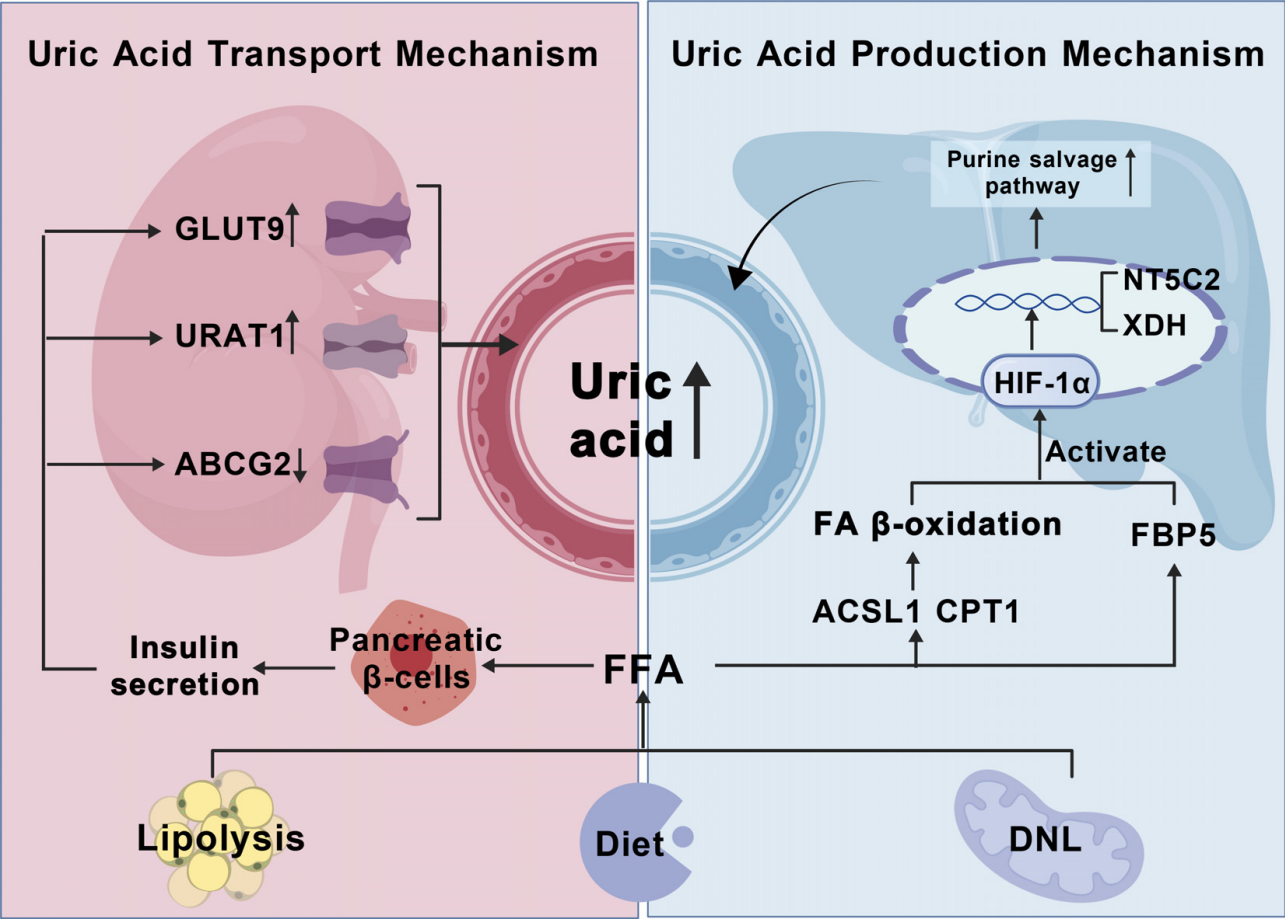
Fatty acid metabolic dysregulation induces hyperuricemia in gout pathogenesis. HIF-α, Hypoxia-inducible factor-1α; NT5C2, 5’-nucleotidase II; XDH, xanthine dehydrogenase; ACSL1, acyl - CoA synthetase long chain family member 1; CPT1, Carnitine Palmitoyltransferase 1; (1) FFA sources: high-fat diet, lipolysis of adipose tissue, ab initio fatty acid synthesis; (2) FAO induces HIF-α transcription, nuclear translocation, and promotes uric acid production by the purine salvage pathway (3) FFA promotes GLUT9,URAT1 uric acid reabsorption proteins through pancreatic islet β-cells, and inhibits ABCG2 uric acid excretory proteins to raise uric acid levels.

FAs may also increase uric acid levels by inhibiting the excretion process. Excessive FAs in the circulatory system promote insulin secretion from pancreatic beta cells, upregulate the expression of renal uric acid reabsorption proteins Glucose transporter type 9 (GLUT9) and urate transporter 1 (URAT1), and reduce the level of uric acid excretion protein ATP-binding cassette transporter G2 (ABCG2), resulting in elevated serum uric acid levels (16, 17, 19). Therefore, it may be inferred that fatty acid metabolism also plays a crucial role in promoting the increase in GA uric acid levels (**Figure 4**).

## 5 Bench to bedside: fatty acid metabolism modulation for gout therapy

### 5.1 Regulation of lipolysis and fat synthesis

#### 5.1.1 PPARγ agonists

PPARγ utilizes both exogenous and endogenous FAs as substrates to promote lipogenesis and lipid synthesis. PPARγ is expressed in the white adipose tissue, the liver, skeletal muscle, the gut, and immune cells, and can effectively reduce the FA levels in the body (108). PPARγ has also been found to exert anti-inflammatory effects and potential protective effects in animal models of neurological, cardiovascular, and psychiatric disorders. Therefore, PPARγ agonists may serve as potential therapeutic agents for treating GA by regulating the FA levels.

Early clinical trials have revealed that ibuprofen can be used to treat acute GA, and no adverse reactions have been reported (109). Recent studies have indicated that ibuprofen may also function as a PPARγ agonist (110). Therefore, curcumin may be the most promising drug currently for the clinical treatment of GA episodes caused by excessively high FA levels. A clinical trial investigating the effects of curcumin on psychological status, inflammation, and oxidative damage markers in patients with type 2 diabetes and coronary heart disease (IRCT20170513033941N63) revealed reduced malondialdehyde (MDA) levels, increased total antioxidant capacity and glutathione (GSH) levels, and upregulated PPAR-γ gene expression in these patients (111). Leriglitazone can improve the pathological state of the disease through dual mechanisms of antioxidant action and agonist effects; indeed, it is an effective agonist of PPARγ. In all the clinical studies, it demonstrated overall good tolerability, with no significant safety findings reported. Lefiglitazone not only promotes FA storage by activating PPARγ but also exerts anti-inflammatory effects by modulating leukotriene synthesis and the expression of the proinflammatory factors TNF-α and IL-6 in patients (112); Pioglitazone is a PPARγ agonist. *Post hoc* analysis of its placebo-controlled trial in patients with non-alcoholic steatohepatitis (NCT00227110) demonstrated that pioglitazone reduces hepatic/visceral fat and improves necrosis and inflammation (113); Aleglitazar, a dual PPAR-α/γ agonist, not only regulates FA storage but has also been clinically demonstrated to have the ability to redistribute fat and reduce hepatic lipotoxicity (114). However, one of aleglitazar’ phase 3 trials (NCT01042769) was terminated because of cardiovascular issues.

Furthermore, numerous natural drug components that exert regulatory effects on PPARγ and can serve as PPARγ agonists have been identified in basic experimental studies. Magnolol is one such natural product derived from *Magnolia officinalis*, which acts as a PPARγ agonist and suppresses inflammation through the PPARγ/NF-κB signaling pathway. Naringenin exerts dual effects by regulating both FA levels and inflammation (115); Naringenin is a flavanone that is primarily found in citrus fruits and enhances adipogenesis through the activation of PPARγ (116); Pectolinarigenin (PEC), a bioactive compound isolated from the Chinese medicinal herb Dajitan, has anti-inflammatory, antioxidative, and anticancer properties, and it also activates PPARγ and enhances ferroptosis through the PPARγ/GPX4 signaling pathway (117); Pollenin B activates PPARγ and alleviates inflammatory responses by regulating the AA metabolism and the JAK-STAT signaling pathway through PPAR (118). Conversely, quercetin (Q), which is a naturally occurring flavonoid, has been demonstrated to have immunomodulatory functions. PPAR-α agonists inhibit NF-κB activation and suppress the JAK/STAT pathway to reduce the expression and release of IL-1β, IL-6, IL-8, and TNF-α, thereby exerting anti-inflammatory effects (119). Furthermore, during the process of cancer and tumorigenesis, IL - 6/JAK/STAT3 promotes fatty acid metabolic reprogramming, epigenetic dysregulation, and the occurrence of cancer and tumors through the activation of the IL - 6/JAK/STAT3 pathway (120); Essential oil from *Fructus alpinia* zerumbet reduces the ubiquitin-mediated degradation of PPARγ and directly binds to the PPARγ protein, thereby increasing its stability (121); Arctigenin, an indirect agonist of PPARγ, activates it through the AMPK/PPARγ pathway (122); Natural products such as asiatic acid (123), total ginsenosides, ursolic acid (124), dillapiole (125) and notoginsenoside R1 (126) can activate PPARγ and regulate FA levels, as detailed in **Table 1**.

**Table 1:** PPARγ agonists.

Drug	Disease	Key targets	Reference
Ibuprofen	GA	PPARγ	(109)
Curcumin	Type 2 Diabetes Mellitus	PPARγ, MDA, GSH, TAC	(111)
Leriglitazone	adrenoleukodystrophy	PPARγ, TNF-α, IL-6, LTB4	(112)
pioglitazone	Non-alcoholic fatty liver disease	PPARγ	(113)
Aleglitazar	Type 2 Diabetes Mellitus	PPARγ	(114)
Magnolol	Diabetic peripheral neuropathy	PPARγ, PPARγ/NF-κB	(115)
Naringenin	PPARγ agonist	PPARγ	(116)
Pectolinarigenin	Intestinal mucositis	PPARγ, PPARγ/GPX4	(117)
pollenin B	Asthma	PPARγ, JAK-STAT, AA	(118)
Essential oil from Fructus Alpinia zerumbet	Atherosclerosis	PPARγ	(121)
Arctigenin	Liver fibrosis	PPARγ, AMPK/PPARγ	(122)
Conversely, quercetin	Inflammation	PPARγ, NF-κB, JAK/STAT	(119)
Total ginsenosides	Alzheimer’s disease	PPARγ	(242)
Ursolic acid	Chemotherapy-induced peripheral neuropathy	PPARγ	(124)
dillapiole	Diabetic nephropathy	PPARγ	(125)
Notoginsenoside R1	Alzheimer’s disease	PPARγ	(126)

#### 5.1.2 Inhibits lipolysis

Inhibiting lipolysis is an effective way to reduce the level of serum FA. Adipose triglyceride lipase (ATGL) and HSL are two key enzymes that inhibit lipolysis. Allopurinol and febuxostat, as xanthine oxidase inhibitors, are medications used to decrease uric acid levels. However, recent studies utilizing serum metabolomics analysis in GA patients have revealed that allopurinol and febuxostat, while lowering uric acid levels, can also alleviate inflammatory responses in GA patients by reducing serum FA levels (97). Therefore, patients taking allopurinol and febuxostat treat GA through synergism: reducing FA levels to suppress inflammatory responses and inhibiting xanthine oxidase to lower uric acid levels. Patients in the intermission phase of GA may benefit from taking this medication to reduce recurrent episodes of GA, which holds significant clinical importance; Acipimox, an HSL inhibitor, significantly reduced FA levels in patients without affecting insulin-stimulated glucose uptake, as observed in a 6-month randomized placebo-controlled trial (NCT01488409) (127); Molecular docking and pharmacological validation revealed that phillyrin inhibits the enzymatic activity of ATGL and suppresses lipolysis (128); Masoprocol, a lipoxygenase inhibitor isolated from the creosote bush, has been shown to decrease adipose tissue lipolytic activity both *in vivo* and *in vitro*. The inhibition of HSL phosphorylation reduces HSL activity, thereby suppressing adipose tissue breakdown and FA levels (129). NG497 is the first human-specific ATGL small-molecule inhibitor that targets the patatin-like domain of human ATGL enzyme activity, eliminating the hormone-stimulated FA release in adipocytes (130).

Research has shown that drugs such as atglistatin (131), puerarin (132), nuciferine (133) and formononetin (134) improve the related diseases by inhibiting fat lipolysis through ATGL and HSL (135–138), as detailed in **Table 2**.

**Table 2:** Drugs that inhibit lipid catabolism.

Drugs	Disease	Key targets	Reference
Atglistatin	Acute pancreatitis	ATGL-specific inhibitor	(131)
Acipimox	Cardiac dysfunction	Inhibits fat breakdown	(243)
Puerarin	Alcoholic liver disease	Inhibiting ATGL activation and HSL phosphorylation to reduce lipolysis	(132)
Nuciferine	Obesity	Reduce HSL and ATGL mRNA expression	(133)
Formononetin	Colitis-associated colon cancer	Reduce ATG and FFA levels, inhibit HSL expression	(134)
Phillyrin	Obesity	Inhibition of ATGL expression	(138)
Cajanotone B and Cajanotone C	Obesity	Inhibition of HSL and ATGL expression	(137)
Bufalin	Breast cancer	Inhibition of HSL expression	(136)
Mulberry1-deoxynojirimycin	Resistin is upregulated in obese	Inhibition of HSL enzyme activity	(135)
NG-497		ATGL competitive inhibitor	(130)

### 5.2 Inhibition of scratch fatty acid synthesis

DNL is a process through which organisms synthesize fatty acids from scratch using non-fat precursors, such as acetyl-CoA, produced through carbohydrate metabolism. Carbohydrates undergo a series of enzymatic reactions and are converted to fatty acid synthesis substrates; first, they convert to acetyl coenzyme A in the presence of ATP-citrate lyase (ACL), which is the first step in endogenous fatty acid synthesis (139), and then, acetyl-CoA carboxylase (ACC) acts to produce malonyl-CoA, thereby initiating DNL. Malonyl-CoA is the main carbon source used for endogenous fatty acid synthesis (140). Fatty acid synthase (FAS) sequentially uses malonyl-CoA to extend the growing fatty acyl chain by two carbons to form the 16-carbon saturated fatty acid palmitate, which is the main product of fatty acid synthesis (141). ACL, ACC, and FAS are the key regulatory steps in endogenous fatty acid synthesis (142), and inhibition of the core enzymes of DNL to reduce FA levels is an attractive therapeutic strategy. Despite challenges in terms of efficacy, selectivity, and safety, several classes of novel synthetic DNL inhibitors are currently in the clinical stage of development and may form the basis of a novel class of therapies (142).

Bempedoic acid is a small-molecule first-in-class of inhibitors of ATP citrate lyase. The therapeutic effect is achieved by reducing FA and cholesterol synthesis through the inhibition of ACLY (143). Firsocostat and cilofexor are orally administered inhibitors that are used to target ACC in clinical drug development for the treatment of steatohepatitis associated with metabolic dysfunction (144). Denifanstat for the treatment of metabolic dysfunction-associated steatohepatitis has been tested in a multicenter, double-blind, randomized, placebo-controlled Phase 2b trial conducted at 100 clinical sites in the U.S., Canada, and Poland, and the results of this Phase 2b trial support the advancement of denifanstat into Phase 3 development (145). In addition, a large number of natural products, such as flavonoids isoginkgetin (146), Morusin (147), nobiletin (148), Hyperoside (149), Formononetin (134), Kaempferol, Kaempferide (150), Myricitrin (151), Delphinidin-3-sambubioside (152), Terpenoids Dehydrocostus lactone (153), Saikosaponin A and D (154), Betulinic acid (155), Phenols Salidroside (156), Gallic acid (157), Gastrodin (158), Sterols Withaferin A (159), Carotenoids lycopene (160) and natural polysaccharides Sargassum pallidum polysaccharide (161) and The alkaloids Berbamine (162), Oxymatrine (163) can also inhibit the synthesis of DNL for therapeutic purposes (164–171), and the specific drugs and their effects are shown in **Table 3**.

**Table 3:** Drugs that inhibit synthesis of derived fatty acids.

Drug	Disease	Key targets	Reference
Isoginkgetin	Atherosclerotic cardiovascular disease	inhibits ACLY activity	(146)
Dehydrocostus lactone	Gastric cancer	promote ACLY ubiquitination	(153)
Morusin	Hepatocellular carcinoma	reduced the ACLY expression and inhibited ACLY activity	(147)
Extract of Pouzolzia zeylanica		ACLY inhibitor	(171)
Aclysiran	Hypercholesterolemia and atherosclerosis	Inhabited the ACLY expression	(170)
Nobiletin	Gastric cancer	Inhabited expression levels of ACLY, and FASN	(148)
Withaferin A	Mammary Tumors	Inhabited expression levels of ACLY, ACC1 and FASN	(159)
Hyperoside	Non-alcoholic fatty liver disease	Inhibited ACLY expression	(149)
Lycopene	Hyperlipidemia	Inhibited ACLY and FAS activity	(160)
Sulforaphane	Non-small-cell lung cancer	Inhabited expression levels of ACLY and FASN	(169)
Formononetin	Colitis-associated colon cancer	Inhabited the ACLY expression	(134)
Salidroside	Atheroscleros	Inhabited the ACLY expression	(156)
Qiwei Jinggan Ling	Alcoholic liver disease	Inhibited FASN and ACC-1 expression	(168)
Schisandrin B	Non-alcoholic liver disease	Inhibited FASN and ACC expression	(167)
Sargassum pallidum polysaccharide	Obesity	Reduce the level of ACC1 and FAS.	(161)
Dihydroartemisinin	Colorectal cancer	Inhibited FAS and ACC expression	(166)
Berbamine	Non-alcoholic fatty liver disease	Reduce the level of ACC and FAS.	(162)
Orange essential oil	Non-alcoholic fatty liver disease	Inhibited ACC expression	(165)
Gallic acid	Non-alcoholic fatty liver disease	Reduce the level of ACC and FASN.	(157)
Gastrodin	Fulminant hepatitis	reduced the expression of ACC phosphorylation	(158)
Kaempferol and Kaempferide	Non-alcoholic fatty liver disease	Inhibited FAS expression	(150)
Saikosaponin A and D	Obesity	Inhibited FAS expression	(154)
Betulinic acid	Nonalcoholic fatty liver disease	Inhibited FAS expression and activity	(155)
Desmodium caudatum extracts	Diabetic nephropathy	Inhibited FAS expression	(164)
Oxymatrine	Non-alcoholic fatty liver disease	increased the levels of ACC and FAS	(163)
Myricitrin	Type 2 Diabetes	Inhibited FAS activity	(151)
Delphinidin-3-Sambubioside	Obesity	Inhibited FASN and ACC mRNA expression	(152)

### 5.3 Inhibition of free fatty acid key target TLRs

FAs promote the development of GA and trigger the initiation of inflammation, mainly by activating its downstream key targets TLR2/TLR4 (172, 173). Therefore, the development of drugs for the treatment of GA through the modulation of TLRs is important. For example, luteolin, a plant-derived flavonoid, downregulates the proinflammatory cytokines in macrophages and inhibits the production of nitric oxide and pro-inflammatory arachidonic acid by blocking the nuclear factor κB (NF-κB) signaling pathway (174). In the acute GA model, luteolin inhibited the expressions of TLR2 and TLR4 mRNAs, reduced their protein expression levels, and ameliorated acute inflammation in GA by downregulating the TLR/MyD88/NF-κB signaling pathway, which in turn inhibited the expression of the downstream inflammatory factors IL-1β, IL-6, and TNF-α (175). Ampelopsis grossedentata total flavonoids also exerted therapeutic effects on GA through the TLR4/MyD88/NF-κB inflammatory pathway in a combined GA model of hyperuricemia (176).

In addition to the drugs that ameliorate inflammation in GA by targeting TLRs, sparsolonin B inhibited FA-induced macrophage inflammation in an osteoarthritis model by selectively blocking the TLR4/MD-2/NF-κB axis (177). Modulation of TLR2/TLR4 by numerous other drugs, such as gastrodin, ferulic acid, sanziguben, cycloastragenol, and taraxasterol, to improve inflammation has been studied and experimentally validated (178–209). All of these factors can improve inflammation through TLR2/TLR4. Inflammation and specific drug information and details are provided in **Table 4**.

**Table 4:** Modulation of TLR2, TLR4 drugs.

Drug	Disease	Key targets	Reference
Ferulic acid	Sciatica causes intense pain	TLR4/NF-κB pathway	(209)
A Novel Drug Combination of Mangiferin and Cinnamic Acid	Rheumatoid arthritis	TLR4/NFκB/NLRP3	(208)
Sanziguben	Diabetic nephropathy	TLR4/NF-κB/NLRP3	(207)
Buyang Huanwu Decoction (BYHWD)	Myocardial infarction	TLR4	(206)
Cycloastragenol	Cecal ligation and puncture (CLP)-induced systemic inflammation in mice	TLR4	(205)
Methyl 3-Bromo-4,5-dihydroxybenzoate	Inflammatory bowel disease	TLR/NF-κB	(204)
Roflupram	Noisy tinnitus	TLR4/NF kB/NLRP3 protein/caspase-1/IL-1 β signaling pathway	(203)
4-Octyl itaconate	Sepsis	TLR4/MAPK/NF-κB	(202)
Pinostrobin	Endotoxemia	TLR4/MD2	(201)
Sanzi Yangqin Decoction	Acute lung injury	TLR2/NF-κB/NLRP3	(200)
Shenshuaifu Granule	Acute kidney injury	TLR4/MyD88/NF-κB pathway	(199)
Taraxasterol	Immune liver injury and alcoholic liver injury	TLR4/MyD88/NF-κB	(198)
Guanmaitong granule	Atherosclerosis	TLR4/MyD88/NF-κB pathway	(197)
Tomentosin	Cerebral ischemia/reperfusion induced inflammatory	TLR4/NLRP3 signalling pathway	(196)
Ginsenoside Rg1	Ochratoxin A-induced liver inflammation	TLR4/NF-κB pathway	(195)
Poria cocos polysaccharides (PCP)	Atherosclerosis	TLR4/NF-κB pathway	(194)
Pterostilbene	Alzheimer’s disease	TLR4/MD2	(193)
Disulfiram	Parkinson’s disease	TLR4/MD2	(192)
Epigallocatechin gallate	Intestinal barrier damage and inflammation	TLR4-NF-κB/MAPKs-NLRP3 pathway	(191)
Licochalcone A	Acute lung injury/Acute respiratory distress syndrome	TLR4/MD2	(190)
Pomegranate polyphenol punicalin	Alzheimer’s disease	TLR4-NF-кB pathway	(189)
Apilarnil	Sepsis	TLR4/NF-κB signaling pathway	(188)
Cyclophosphamide	Bladder dysfunction	TLR4/NF-κB signaling pathway	(187)
CycloZ	Asthma	TLR4	(186)
Astilbin	Sepsis	TLR4/NF-κB	(185)
Corilagin	Sepsis	TLR4/MyD88	(184)
Involucrasin B	Asthma	TLR4-NF-κB-NLRP3	(183)
Ruscogenin	Ruscogenin	TLR4/MyD88	(182)
Chlorogenic acid	Kidney fibrosis	TLR4/NF-κB	(181)
Icariin	Diabetic nephropathy	TLR4/NF-κB	(180)
Phillygenin	Diabetic nephropathy	TLR4/MyD88/NF-κB	(179)
Gastrodin	Alzheimer’s disease	TLR4/TRAF6/NF-κB	(178)

### 5.4 Regulation of arachidonic acid metabolism

Abnormal AA metabolism plays an important role in GA episodes by recruiting neutrophils to the inflammatory site and thereby promoting the inflammatory flare-ups and their persistence; therefore, exploring the development of drugs to improve GA by modulating AA metabolism is worthwhile.

#### 5.4.1 COX-2 inhibitor

Etoricoxib is a selective cyclooxygenase-2 (COX-2) inhibitor, which was evaluated in a randomized, double-blind, active comparator-controlled trial for the treatment of acute GA. Research has shown that etoricoxib rapidly and selectively inhibits COX-2, and its efficacy in terms of treating various clinical manifestations of GA, including pain and inflammation, is comparable to that of indomethacin (210); Rofecoxib is a selective COX-2 inhibitor, which was evaluated in clinical trials for acute GA, and it was reported to reduce the expression of inflammatory mediators, such as IL-6, and alleviate patient pain by inhibiting COX-2-regulated AA metabolism (211); Celecoxib is a COX-2 inhibitor. The clinical trial for acute GA (NCT00549549), with a randomized, double-blind, double-dummy, active-controlled design, was conducted across 100 centers. The results indicated that monitoring the treatment response throughout the entire acute episode until complete resolution is clinically significant. The efficacy of high-dose celecoxib (800/400 mg) was comparable to that of indomethacin (50 mg tid). Furthermore, the duration of pain relief achieved using high-dose celecoxib appeared to be longer than that achieved with indomethacin (212); etodolac (a COX-2 inhibitor) demonstrated analgesic effects in clinical trials for general anesthesia (213).

Additionally, basic research has identified numerous drugs capable of inhibiting COX-2. Leonurine, for example, exerts its anti-GA effects by suppressing COX-2 and LOX-5 expression, thereby reducing the prostaglandin E2 (PGE2) and LTB4 levels in synovial macrophages and diminishing neutrophil recruitment (214); The whole plant extract of *Fimbristylis aestivalis*, which contains rosmarinic acid, catechin hydrate, and syringic acid, has inhibitory effects on COX-2 and effectively alleviates inflammatory responses (215); Thymol-1,5-disubstituted pyrazole hybrids and novel 5,6-diphenyl-1,2,4-triazine-3-thiol derivatives exhibit inhibitory potential against both COX-2 and 5-LOX enzymes and possess anti-inflammatory properties (216, 217); Resveratrol amide derivatives used as selective COX-2 inhibitors in molecular docking studies demonstrated partial entry into the 2°-pocket of the COX-2 active site and interaction with the amino acid residues responsible for COX-2 selectivity, which have orientation and binding interactions similar to Rofecoxib (218). Nimesulide (219), Vismodegib (220), New Pyrimidine-5-Carbonitriles (221), Sulfonamide-Pyrazole derivatives (222) and lariciresinol (223) exert regulatory effects on arachidonic acid metabolism by inhibiting COX-2, the specific drugs used are shown in **Table 5**.

**Table 5:** COX-2 inhibitors.

Drug	Disease	Key targets	Reference
Fimbristylis aestivalis	inflammation	COX-2	(215)
Novel 5,6-diphenyl-1,2,4-triazine-3-thiol derivatives	inflammation	COX-2,LOX-5	(217)
thymol - 1,5-disubstitutedpyrazole hybrids	inflammation	COX-2,LOX-5	(216)
resveratrol amide derivatives		selective COX-2 inhibitor	(218)
Nimesulide	cancer	COX-2 inhibitor	(219)
Leonurine	Gouty arthritis	COX-2,LOX-5	(214)
Vismodegib		COX-2 inhibitor	(220)
New Pyrimidine-5-Carbonitriles		COX-2 inhibitor	(221)
Sulfonamide-Pyrazole derivatives		COX-2 inhibitor	(222)
lariciresinol		selective COX-2 inhibitor	(223)
etoricoxib	Gouty arthritis	COX-2 inhibitor	(210)
Rofecoxib	Gouty arthritis	selective COX-2 inhibitor	(211)
Celecoxib	Gouty arthritis	COX-2 inhibitor	(212)
Etodolac	Gouty arthritis	COX-2 inhibitor	(213)

#### 5.4.2 LOX-5 inhibitor

The Chinese medicine Huzhen Tongfeng Formula (HZTF) is composed of four Chinese herbs: Polygoni Cuspidati Rhizoma et Radix (PCRR, the root and rhizome of Polygonum cuspidatum Sieb. et Zucc.), Ligustri Lucidi Fructus (LLF, the fruit of Ligustrum lucidum Ait.), Herba Plantaginis (HP, the dried whole grass of Plantago asiatica L.), and Nidus Vespae (NV, the honeycomb of Polistes olivaceus (De Geer), Polistes Japonicus Saussure, or Parapolybiavaria Fabricius) (224). In the GA model, HZTF attenuated leukocyte recruitment in the synovium by significantly inhibiting the lipoxygenase pathway of AA for the treatment of GA (224). Leucas zeylanica, which is used in traditional medicine for the treatment of GA, can also improve GA inflammation by reducing the metabolite levels through the inhibition of the AA lipoxygenase pathway (225).

In addition, many other drugs can improve inflammation by reducing neutrophil recruitment through the inhibition of this pathway (225). For example, zileuton (226), red-kerneled rice proanthocyanidin (227), malabaricone C (228), caffeic acid (89), lysionotin (229) and magnolin (230) inhibit AA lipoxygenase by inhibiting the expression of key enzymes or enzyme activity reduction to achieve the anti-inflammatory effects (231–236), the specific drugs used are shown in **Table 6**.

**Table 6:** Drugs that inhibit 5-LOX.

Drug	Disease	Key targets	Reference
Zileuton	Subarachnoid hemorrhage	Inhibits 5-LOX expression, Reduces leukotriene synthesis	(226)
Red-kerneled rice proanthocyanidin	Psoriasis	Inhibits 5-LOX expression, Reduces leukotriene synthesis	(227)
Malabaricone C	Psoriasis	Inhibition of 5-LOX activity.	(228)
ALR-38	Inflammation	Inhibition of 5-LOX	(236)
ALR-6 and ALR-27	Inflammation	Suppression of LT generation by FLAP/5-LOX	(236)
Caffeic acid	Sepsis	Inhibits 5-LOX expression and reduces LTB4 levels.	(89)
lysionotin	Glioma	Inhibition of 5-LOX and downstream LTB4 receptor expression	(229)
Magnolin	Inflammatory bowel disease	Inhibition of 5-LOX expression	(230)
*δ*-tocotrienol and *γ*-tocotrienol	Inflammation	Competitively inhibits 5-LOX activity	(235)
7-Hydroxyflavone	Neuropathic pain	Binds to the ligand binding site of 5-LOX and inhibits the action of 5-LOX	(234)
Pygeum africanum	Benign prostatic hyperplasia	Reduces leukotriene production	(233)
Compound Dahuang Baiji spray	Acute radiodermatitis	Inhibited the expression of 5-LO	(232)
Astragaloside IV	Non-alcoholic fatty liver disease	Inhibited the expression of 5-LO and LTB4	(231)

This paper systematically reviews the regulation of GA metabolism and immune responses in the hyperuricemia occurring due to FA metabolic disorders and identifies targets for personalized therapy based on the underlying mechanisms (210, 211). Therefore, the use of AA-related enzyme inhibitors to treat acute GA patients represents a reliable therapeutic strategy. Second, PPARγ agonists, the drugs that lower FA levels by promoting fat synthesis, are expressed in adipose tissue, the liver, skeletal muscle, and immune cells (108). This characteristic renders PPARγ agonists potentially applicable to patients with GA and hyperuricemia associated with cyclic FA disorders (30). However, the use of this medication may increase body weight and should be used with caution in patients with GA who are also obese or have insulin resistance. Third, HSL and ATGL inhibitors are used (127, 128), which reduce FA levels by suppressing lipolysis and do not selectively regulate specific types of FAs, and may, therefore, be applicable for the prevention and treatment of patients at risk of elevated circulating FA levels. Fourth, TLR inhibitors not only block fatty acid-induced inflammatory activation but also inhibit the TLR signaling associated with MSU crystals (45), thereby exerting a more comprehensive anti-inflammatory effect. These drugs may, during the course of GA, promote the resolution of inflammation and finally reduce the free FA levels by inhibiting key enzymes involved in fatty acid synthesis, such as FASN, ACC, and ACL. Moreover, palmitoylation during fatty acid synthesis influences the key inflammatory pathways, such as TLR/MyD88 (45) and NLRP3 inflammasome activation and assembly (55, 56). Therefore, such inhibitors may be more suitable for the resolution of GA-related inflammation.

Disordered FA metabolism further promotes inflammation, and elevation of the uric acid levels, primarily through high levels of FAs, activates the initiation of inflammation. The uric acid is taken up by cells as a substrate for FAO; moreover, abnormal AA metabolism contributes to the eruption and persistence of acute inflammation in GA. Therefore, by organizing and summarizing the drugs that modulate the above mechanisms, a basis for subsequent GA treatment and novel drug development is prepared.

## 6 Discussion and perspectives

GA is a metabolic-inflammatory disorder characterized by an intricate crosstalk between aberrant FA metabolism and immune dysregulation (237). Emerging evidence underscores the centrality of FA metabolism perturbations in driving disease progression, from hyperuricemia-triggered crystal deposition to sustained NLRP3 inflammasome activation, ultimately forming a self-perpetuating “metabolism-immunity” axis.

### 6.1 FA overload as a pathogenic catalyst

Elevated levels of circulating FAs, derived predominantly from adipose lipolysis, DNL, and excessive dietary intake (238), represent the critical pathogenic drivers of GA. Mechanistically, FAs exacerbate inflammation through three synergistic pathways. First, FAs synergize with monosodium urate (MSU) crystals to amplify macrophage activation through TLR2/MyD88/NF-κB and TLR4-MD2/MyD88/NF-κB signaling, leading to NLRP3 inflammasome assembly and IL-1β maturation (41). represent the critical pathogenic drivers of GA. Mechanistically, FAs exacerbate inflammation through three synergistic pathways. First, FAs synergize with MSU crystals to amplify macrophage activation through TLR2/MyD88/NF-κB and TLR4-MD2/MyD88/NF-κB signaling, leading to NLRP3 inflammasome assembly and IL-1β maturation (17, 57, 58). In addition, fatty acid metabolic reprogramming in GA drives distinct pathways: β-oxidation in hepatocytes and macrophages promotes urate synthesis and proinflammatory cytokine release (18, 64, 93), AA metabolism in neutrophils drives the LTB4-BLT1-mediated chemotaxis and NETosis, amplifying local inflammation (76–78).

### 6.2 Therapeutic implications and challenges

Despite the clinical correlations between FA dysmetabolism and GA severity, mechanistic insights remain fragmented, with most studies limited to biomarker associations rather than causal validation. This study identified three promising therapeutic strategies for targeting FA metabolism. First is the urate-lowering agents allopurinol and febuxostat, which are traditional clinical agents used to inhibit xanthine oxidase and reduce uric acid levels. Recent studies have revealed that these agents also exert a regulatory effect on FA levels in patients with FA-related GA. Although the precise mechanism remains unclear to date, these drugs hold potential as therapeutic options for GA patients with FA metabolism disorders (97). Second, metabolic enzyme inhibitors, which are the COX-2 inhibitor nonsteroidal anti-inflammatory drugs used in clinical trials to treat patients with GA, can target specific AA metabolic pathways as well as alleviate inflammation during acute GA episodes. These are, therefore, particularly suitable for patients identified through a lipidomic analysis as having AA metabolic dysfunction (210, 211). Third, the multitarget natural compounds curcumin, resveratrol, and berberine exhibit pleiotropic benefits by suppressing FA generation, blocking AA-derived eicosanoid synthesis, and enhancing insulin sensitivity, with favorable safety profiles (107, 146, 181).

However, clinical translation faces numerous challenges. First, there may be off-target effects from the systemic modulation of the FA pathway. For example, the nonsteroidal anti-inflammatory drug fenoprofen exerts its anti-inflammatory action by inhibiting prostaglandin (PG) biosynthesis through COX blockade. However, it also acts non-selectively on both COX-1 and COX-2 isoforms, leading to numerous side effects and off-target effects (239). Second, the tissue-specific differences in FA metabolism, where FA metabolism in various organs, such as the liver and kidneys, influences distinct stages of GA progression, and the varying tissue affinities exhibited by drugs, such as the PPARγ agonists pioglitazone (113) and aleglitazar (240), exhibit stronger affinity for the hepatic tissue, redistributing visceral fat to peripheral tissues. Whether this would induce adverse reactions in the pathological state of GA warrants further investigation. Additionally, the long-term safety concerns associated with single-pathway metabolic modulators must be considered. Drugs typically require multi-pathway regulation for the management of disease progression; however, the prolonged inhibition of a single pathway may lead to compensatory enhancement of other pathways. Therefore, the use of such drugs should be limited to short-term therapeutic interventions, and whether the side effects associated with long-term use are manageable must be carefully evaluated.

### 6.3 Future research directions

While this review synthesizes current evidence, critical knowledge gaps nonetheless persist. Neutrophil-FA dynamics: How FA metabolism regulates neutrophil plasticity (e.g., N1/N2 polarization) during GA flares remains unexplored. Spatiotemporal resolution: Advanced techniques (single-cell RNA-seq and spatial metabolomics) can be used to map FA flux heterogeneity across synovial cell populations. Preclinical models: Humanized GA models incorporating dietary/metabolic stressors are needed to recapitulate FA-immune interactions (241).

This study is, to the best of the author’s knowledge, the first systematic integration of FA metabolic derangements with GA pathogenesis and therapeutic development. This work, by delineating the “FA-AA-inflammasome” axis and cataloging the mechanism-based interventions, provides a framework for developing precision therapies that simultaneously address metabolic dysfunction and sterile inflammation in GA.
